# Characterizing the Contaminant-Adhesion of a Dibenzofuran Degrader *Rhodococcus* sp.

**DOI:** 10.3390/microorganisms13010093

**Published:** 2025-01-06

**Authors:** Yu Chen, Qingquan Wei, Xudi Wang, Yanan Wu, Changai Fu, Xu Wang, Hangzhou Xu, Li Li

**Affiliations:** 1 Shandong Provincial Key Laboratory of Water Pollution Control and Resource Reuse, School of Environmental Science and Engineering, Shandong University, Qingdao 266237, China; chenyu010611@163.com (Y.C.); 17854172473@163.com (Q.W.); wangxudi1999@163.com (X.W.); 201920359@mail.sdu.edu.cn (Y.W.); 201812484@mail.sdu.edu.cn (C.F.); 201820330@mail.sdu.edu.cn (X.W.); 2 Shandong Provincial Engineering Center on Environmental Science and Technology, Jinan 250061, China

**Keywords:** adhesion mechanism, bacteria-adhesive behaviors, extracellular polymeric substances, dibenzofuran, biofilm, surface characteristics

## Abstract

The adhesion between dibenzofuran (DF) and degrading bacteria is the first step of DF biodegradation and affects the efficient degradation of DF. However, their efficient adhesion mechanism at the molecular level remains unclear. Therefore, this study first examined the adhesive behaviors and molecular mechanisms of *Rhodococcus* sp. strain p52 upon exposure to DF. The results showed that the adhesion between strain p52 and DF is mediated by extracellular polymeric substances (EPSs). Compared with sodium acetate as a carbon source, the percentages of glucose and proteins related to electron transfer, toxin–antitoxin, and stress responses were elevated, which were analyzed by polysaccharide composition and proteomics, and the contents of extracellular polysaccharides and proteins were increased. Moreover, biofilm analysis suggested an increase in EPS content, and the change in components increased biofilm yield and promoted loose and porous aggregation between the bacteria; this aggregation caused an increase in the specific surface area in contact with DF. The surface characteristics analysis indicated that the production of EPS reduced the absolute value of the zeta potential and increased the hydrophobicity of strain p52, which was beneficial for the adhesion of strain p52 and DF. These findings help us to enhance the understanding of the adhesion mechanisms and bioremediation of polycyclic aromatic hydrocarbons by degrading bacteria.

## 1. Introduction

Dibenzofuran (DF) is an oxygen-containing heterocyclic polycyclic aromatic hydrocarbon and a nonhalogenated analog of dioxins. The presence of DF in the environment poses risks to human health and ecosystems [1,2]. DF is mostly produced by anthropogenic processes such as the incomplete combustion of coal and oil. In addition, a small portion is produced by natural processes such as forest fires [3]. Despite the reduction in emissions due to new energy alternatives and control measures, DF is still examined in wastewater samples worldwide (concentrations ranging from 0.5 to 11.7 mg/L) [4]. Furthermore, due to its strong hydrophobicity, the majority of DF adheres to air and soil particles and has low bioavailability [5,6].

Although DF is intractable, some DF-degrading bacteria, such as *Rhodococcus* [7], *Sphingomonas* [8], *Bacillus* [9], and *Pseudomonas* [10], have been reported to be able to degrade DF. The degradation of DF is divided into two steps, namely adhesion and subsequent intracellular aerobic degradation. At present, the intracellular degradation mechanism has been elucidated. The oxidation reaction, which is catalyzed by oxygenase, is first performed. Subsequently, DF is metabolized into various intermediate metabolites and completely degraded through the tricarboxylic acid cycle [11]. However, the mechanism of adhesion between DF and biodegrading bacteria remains unclear; this lack of understanding hinders the further improvement of DF degradation efficiency.

Adhesion between the microorganisms and organic pollutants can effectively reduce the mass transfer distances and thus promote degradation [12]. Adhesion is usually accompanied by bacterial growth and multiplication on the surface of hydrophobic organic compounds, and this process is accompanied by biofilm formation [13]. Moreover, the zeta potential and hydrophobicity of bacteria, which are important surface characteristics of bacteria, are altered to promote the adhesion between the bacteria and hydrophobic pollutants [14]. Extracellular polymeric substances (EPSs), as cell-secreted polymers, are widely distributed on the surface of microbial cells and influence the biofilm formation and the surface characteristics of bacteria, in turn affecting adhesion between the bacteria and contaminants [15]. On the one hand, when exposed to pollutants, bacteria secrete more EPSs (loose EPS and tight EPS), which promotes more biofilm production and affects the zeta potential and hydrophobicity of bacteria, thus favoring bacterial adhesion to the contaminants. Rodrigues et al. [16] reported that *P. aeruginosa* utilized phenanthrene better than fluorene because of the greater EPS secretion by the former strain, accompanied by the increased biofilm yield and hydrophobicity of the strain. On the other hand, even for the same strain, the extracellular polysaccharides and proteins, the main components of EPSs, were secreted by the bacteria change when induced by the pollutants. Specifically, the composition of extracellular polysaccharides and proteins also affected the roles of EPSs in adhesion. Onbasli et al. [17] analyzed the differences in the extracellular polysaccharides of four *Pseudomonas* strains in mineral media with several organic contaminants as a carbon source, respectively, and reported that the proportion of glycerol in the extracellular polysaccharides increased compared with nutrient broth medium.

In addition, EPSs play roles in the protection, anti-toxicity, flocculation, and dissolution of contaminants, in addition to biofilm formation and adhesion [15,18]. The protein component of EPSs is also an important constituent of the extracellular enzymes, which play a role in the degradation of the contaminants. The hydrolysis of the excess EPS components may also affect cellular motility [19]. Although it has shown that the adhesion of DF by bacteria is related to the EPSs secreted by bacteria [9], the relationship between the changes in the EPS composition and content and the changes in the behavior of the bacteria has been less studied during the efficient adhesion process of DF by bacteria.

When DF was used as the sole carbon source, a previously isolated DF-degrading bacterium, *Rhodococcus* sp. strain p52, had the ability to completely degrade 500 mg/L DF within 48 h, and the strain mainly initiated DF metabolism via angular dioxygenation [20]. As a common carbon source in bioaugmented reactors, sodium acetate (NaOAc) was chosen as the nonselective alternate carbon source. Bacterial growth on this compound also influenced the expression of select proteins, including DF dioxygenase [21]. Moreover, as a hydrophilic carbon source, NaOAc can also be compared with DF to help understand the difference in the utilization of hydrophilic and hydrophobic carbon sources by strain p52. This study aimed to investigate the effects of EPSs secreted by strain p52 on the biofilm and surface properties during DF degradation, reveal the mechanism of the efficient adhesion of strain p52 to DF, and analyze the effect of EPSs on DF degradation. This work provides evidence for the mechanism of the EPS-mediated adhesion process and demonstrates that the adhesion behavior enhanced the biodegradation potential, thus providing a strategy to improve the application of polycyclic aromatic hydrocarbon-degrading and heterocyclic polycyclic aromatic hydrocarbon-degrading bacteria in the environment.

## 2. Materials and Methods

### 2.1. Chemicals, Bacterial Strain, and Culture Conditions

All chemicals used in this study were of analytical grade. DF, dibenzo-*p*-dioxin (DD), anhydrous methanol, and acetic acid were purchased from J&K Scientific (Beijing, China). N-hexadecane and ethyl acetate were purchased from China National Pharmaceutical Group Corporation (Shanghai, China). NaOAc and all the other chemicals were obtained from Sangon Biotech Co., Ltd. (Shanghai, China).

*Rhodococcus* sp. strain p52 was obtained from oil-contaminated soil [20] and deposited in the China Center for Type Culture Collection (no. M2011181). This strain was activated in 50 mL Luria–Bertani medium and cultivated for 16 h in a shaker (30 °C and 180 rpm). Strain p52 was harvested, washed three times with a carbon-free mineral medium (CFMM), and resuspended in CFMM to OD_600_ ≈ 1.0 (corresponding to 10^6^–10^7^ colony-forming units (CFUs)/mL) [20]. Then, the OD-adjusted strain p52 suspension was used in all subsequent experiments with 5% (*v*/*v*) inoculum.

### 2.2. Experimental Procedures

#### 2.2.1. Effects of DF as a Carbon Source on Strain p52

To equalize the number of carbon moles, 0.50 g/L of DF and 1.46 g/L of NaOAc were added to 250 mL Erlenmeyer flasks containing 50 mL CFMM, respectively [22]. Then, the OD-adjusted strain p52 suspension was inoculated into 50 mL CFMM at 5% (*v*/*v*) concentration. Then, all flasks were incubated at 30 °C and shaken at 180 rpm for 48 h.

After culturing for 0 h, 12 h, 24 h, 36 h, and 48 h with DF and NaOAc as the sole carbon sources, the utilization of DF and NaOAc and the growth of strain p52 were measured. Moreover, the biofilm yield and essential surface characteristics (zeta potential and hydrophobicity) of strain p52 were determined. After culturing for 24 h, scanning electron microscopy (SEM) images of the strain p52 biofilm were analyzed. The EPSs of strain p52 were extracted, and the polysaccharide and protein yields, functional groups, compositions, and proportions of EPS were analyzed. Furthermore, to further analyze the effect of the carbon source on the biofilm yield, the biofilm yield of strain p52, with DF and DD as the sole carbon sources, was measured after 24 h.

All experiments were carried out in 250 mL Erlenmeyer flasks, with the exception of the measurement of the biofilm yield; this measurement was performed in a 96-well cell culture plate with 200 μL medium per well [23]. The concentrations of the carbon source and added cells were the same as those in the 250 mL Erlenmeyer flask. Strain p52 was cultured under static conditions at 30 °C for 48 h.

#### 2.2.2. Effects of Proteinase K and Lysozyme on Strain p52

To verify the effect of EPSs on strain p52, proteinase K, and lysozyme, 0.7 U/mL for each was added to the cell suspension that was adjusted to OD_600_ ≈ 1.0 [24]. The bacterial suspension was vortexed for 3 min for sufficient mixing, and 5% (*v*/*v*) strain p52 was inoculated into DF-supplemented CFMM (30 °C and 180 rpm). The effects of enzyme treatment on DF degradation and strain p52 biofilm yield were determined.

### 2.3. Analytical Methods

#### 2.3.1. Determination of the DF and NaOAc Concentrations

The residual DF was extracted with an equal volume of ethyl acetate by shaking for 30 min. Then, the mixture was allowed to stand for 30 min, ensuring that the organic and aqueous phases were completely stratified. The concentrations of DF were determined by gas chromatography (GC, 7890B, Agilent, Santa Clara, CA, USA) equipped with an HP-5 column (30 m × 320 µm × 0.25 μm) and a flame ionization detector according to previously described methods [20,25].

The NaOAc concentration was measured using ultraviolet spectrophotometry. First, the maximum wavelength was detected by wavelength scanning, and the details are presented in Figure S1. Then, the content of NaOAc was determined according to the standard curve [26].

#### 2.3.2. Measurement of the Growth of Strain p52

Bacterial growth on DF-supplemented and NaOAc-supplemented CFMM was measured using plate count methodology [27].

#### 2.3.3. Analysis of the Biofilm and Surface Characteristics of Strain p52

To explore the effects of EPSs on the biofilm and surface characteristics of strain p52, the yield and SEM images of strain p52 biofilms were analyzed, and the zeta potential and hydrophobicity were measured. The method from Saotome et al. [28] was improved to measure the biofilm yield, and the details are provided in Text S1. Strain p52 was immobilized on polystyrene sheets of approximately 1 cm × 1 cm, and the morphology of the biofilms was observed using SEM [29].

The zeta potential of strain p52 was measured using a nanometer particle size zeta potential meter (Nano ZSE, Malvern-Passaco, Malvern, UK) [30]. Hydrophobicity was determined using a modified bacteria adherence to hydrocarbons (BATH) method [31], and the details are listed in Text S2. To further determine the hydrophobicity of strain p52, the contact angle between strain p52 and water was determined via the method from Vanloosdrecht et al. [32].

#### 2.3.4. Determination of the Polysaccharide and Protein Contents of Strain p52

To assess the effects of extracellular polysaccharides and protein on the adhesion between strain p52 and DF, EPSs were extracted at 24 h, first. The culture broth was rotary evaporated at 35 °C until it became a viscous liquid. The loose EPS of strain p52 was extracted according to the method from Weather et al. [33], and the tight EPS of strain p52 was extracted via the method from Aguilera et al. [34]. Second, the bacterial precipitate and the extracted EPS were weighed after being freeze-dried. Third, the polysaccharide content of EPSs was determined using anthrone sulfuric acid colorimetry, according to the method from Trevelyan et al. [35]. The protein content in EPSs was determined using the Coomassie Brilliant Blue staining, according to the method from Bradford [36]. Finally, to further determine the existence of polysaccharides and proteins in EPSs, the functional groups of the substances in the samples were determined using a Fourier transform infrared spectrometer (FTIR, Nicolet iS50, Thermo Fisher, Waltham, MA, USA) [37].

#### 2.3.5. Analysis of the Composition and Proportion of Monosaccharide and Protein in EPSs

The monosaccharide composition was determined using ion chromatography (ICS 5000, Thermo Fisher) [38], and the details are listed in Text S3. The protein composition was determined on an UltiMate 3000 RSLCnano system coupled online with a Q Exactive HF mass spectrometer through a Nanospray Flex ion source. The data were analyzed with MaxQuant (1.6.6.0) using the Andromeda database search algorithm and retrieved from the NCBI protein database [39,40]; the details are provided in Text S4.

### 2.4. Statistical Analysis

Statistical analyses were performed using SPSS 26 software. One-way analysis and Student’s *t*-test of variance were performed to compare the significant differences between the treatments at *p* < 0.05. All experiments were performed in triplicate, and the results are expressed as the average ± standard deviation.

## 3. Results and Discussion

### 3.1. Degradation of DF by Strain p52

**Figure 1:**
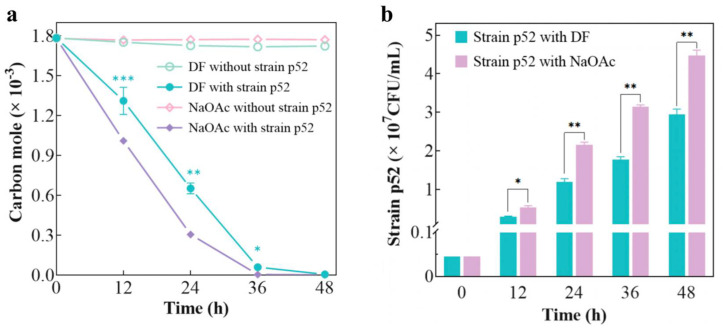
(**a**) Degradation of DF and NaOAc by *Rhodococcus* sp. strain p52 and (**b**) growth of strain p52 with DF and NaOAc as carbon sources (DF: dibenzofuran; NaOAc: sodium acetate). Significant differences between the test and control groups are indicated by asterisks at *p* < 0.05 (*), *p* < 0.01 (**), and *p* < 0.001 (***).

After equalizing the number of carbon moles of DF and NaOAc, the degradation of both compounds by strain p52 was examined (Figure 1a), and strain p52 was able to exhaust carbon sources within 48 h. Furthermore, the consumption of NaOAc was faster than that of DF by strain p52, and the cell number of strain p52 was also greater in NaOAc-supplemented CFMM than in DF-supplemented CFMM (Figure 1b). This result occurred because NaOAc is soluble in water and was more easily utilized by bacteria than hydrophobic DF. The hydrophobicity of hydrophobic organic compounds has been reported to prevent their approach to bacteria. The transport of hydrophobic organic compounds to the intracellular area of bacteria also requires more energy than the transport of hydrophilic organic compounds [41]. In addition, DF is a highly toxic pollutant, and even degradable strains require an adaptive buffer time for its utilization. A previous study suggested that as the degradation proceeds, the surface characteristics and biofilms of the strain change to adhere to the contaminants and bacteria secrete enzymes to degrade the toxic substances [42]. Except for carbon sources, the remaining abiotic factors (such as temperature and pH) and biotic factors (such as microorganism interactions) also influence the efficacy of bioremediation. As demonstrated by previous studies, different microorganisms require distinct optimal temperature and pH conditions for degrading pollutants and, in general, temperatures below 20 °C and strong acid–base properties of the environmental medium are detrimental to biodegradation [43]. Furthermore, diverse interactions between microorganisms such as competition, cooperation, and symbiosis have different effects on bioremediation [11]. The effects of various environmental conditions on bioremediation should be further studied.

### 3.2. Effects of DF on the Biofilm and Surface Characteristics of Strain p52

**Figure 2:**
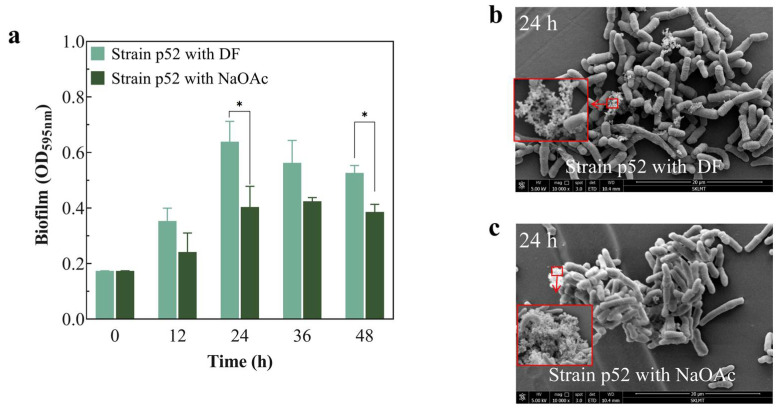
Analysis of biofilms of *Rhodococcus* sp. strain p52. (**a**) Biofilm yield of strain p52. (**b**,**c**) SEM images of strain p52 biofilms at 24 h; the framed diagrams show the EPS on the surface of strain p52 (DF: dibenzofuran; NaOAc: sodium acetate; SEM: scanning electron microscopy; EPS: extracellular polymeric substance). Significant differences between the test and control groups are indicated by asterisks at *p* < 0.05 (*).

To explore the effects of DF on the biofilm formation of strain p52, the biofilm yield was measured when DF and NaOAc were used as carbon sources (Figure 2a). The results showed that the strain p52 biofilm yield increased with incubation time and peaked at 24 h. Thereafter, the strain p52 biofilm yield decreased as exhaustion of the nutrition and disaggregation of the biofilm occurred [44]. When DF was used as the carbon source, the strain p52 biofilm yield was higher than that when NaOAc was used as the carbon source throughout the measured time period. The most evident difference was observed at 24 h, and the difference diminished as DF was degraded. These results indicated that the presence of DF induced strain p52 to produce more biofilm, which allowed for the aggregation of the bacteria. Bacteria usually need time to adapt to toxic substances and upregulate the expression of relevant metabolic enzymes to degrade toxic substances. Biofilms play a buffering role and facilitate resistance to unfavorable external environments [45]. With the expression of metabolic enzymes, the contaminant is metabolized and used as a source of carbon and energy for the bacteria [33]. Measurements of the DF and DD biofilm yields can further confirm this view. Due to the higher toxicity of DD and the lower ability of strain p52 to degrade DD than DF [20], the yield of biofilm produced at 24 h was approximately 1.29 times greater when DD was the sole carbon source than when DF was used (Figure S2).

The cell membrane surface morphology of strain p52 was observed with SEM, and the bacteria all aggregated in both the DF-supplemented (Figure 2b) and the NaOAc-supplemented CFMM (Figure 2c). However, the aggregation degree was different. When DF was used as the carbon source, the bacteria were loosely and porously aggregated; thus, a larger specific surface area was attained and more contact sites with DF were provided, such that the degradation efficiency of DF improved. This was in agreement with the view of loading bacteria onto porous materials to facilitate pollutant degradation [46]. When water-soluble NaOAc was the carbon source, strain p52 was very close together. Shi et al. [47] reported similar results, where sludge structures in the NaOAc group were relatively tight compared with sludge without an additional carbon source. Furthermore, a white floc was found on the cell wall surface of both carbon sources and was presumed to be EPSs attached to the surface of strain p52 (Framed part of Figure 2b,c). Researchers generally believe that cells in biofilms are encapsulated by EPSs and that the biofilm yield is slightly reflective of the yield of EPSs [48]. The increased tolerance and buffer ability of biofilms to toxic pollutants are usually attributed to the EPS matrix [49].

**Figure 3:**
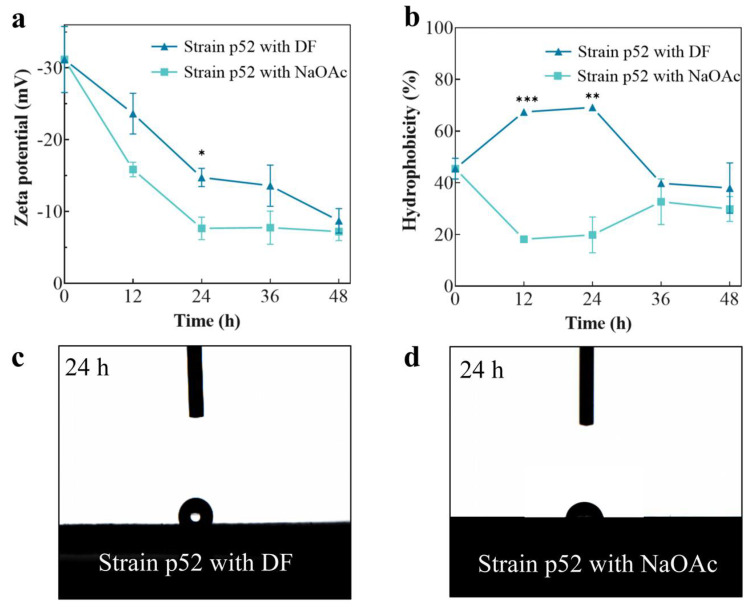
Surface characteristics of *Rhodococcus* sp. strain p52. (**a**) Zeta potential of strain p52. (**b**) Hydrophobicity of strain p52 determined via the BATH method. (**c**,**d**) Contact angle between strain p52 and water at 24 h (DF: dibenzofuran; NaOAc: sodium acetate; BATH: bacteria adherence to hydrocarbons). Significant differences between the test and control groups are indicated by asterisks at *p* < 0.05 (*), *p* < 0.01 (**), and *p* < 0.001 (***).

The zeta potential of strain p52 was measured using DF and NaOAc as the sole carbon source (Figure 3a). When DF was used as the carbon source, strain p52 was negatively charged, and the zeta potential tended to approach zero with increasing incubation time. According to the SEM results (Figure 2b,c), strain p52 produced EPSs at 24 h. Hua et al. [50] reported that the addition of EPSs during the degradation of n-hexadecane by *Enterobacter cloacae* TU reduced the absolute value of the zeta potential. Derjaguin–Landau–Verwey–Overbeek (DLVO) theory, which relates to the relationship between the bilayer interactions and the total Gibbs free energy, indicates that the solution is in an unstable state when the zeta potential approaches zero; these conditions are beneficial for the flocculation of particles and adhesion between bacteria and hydrophobic contaminants [51]. Smith et al. [30] reported that a decrease in the absolute value of the surface potential of *Mycobacterium smegmatis* resulted in increased adhesion of the bacteria to the abiotic phase. Therefore, the absolute value of the zeta potential of strain p52 decreased with the degradation of DF and the production of EPSs, and this was beneficial for increasing the adhesion of strain p52 to DF. When NaOAc was used as the carbon source, the zeta potential of strain p52 was closer to zero than that of DF-supplemented CFMM. These results were consistent with the conclusions of Górna et al. [52]. In their study, compared with that of diesel as a carbon source, the zeta potential of *P. aeruginosa* was closer to zero when glucose was used as a carbon source. The results indicated that adhesion to the contaminant was weaker than that to hydrophilic substrates even for the degrading strain, resulting in a weaker utilization efficiency of bacteria to hydrophobic contaminants. It is related to the fact that the surface of most bacteria has low hydrophobicity, and they are more inclined to adhere to hydrophilic carbon sources. In addition, the toxicity of contaminants makes it necessary to have a buffer time before the adhesion process, during which the bacteria secrete substances and change surface properties to adapt to the contaminants.

The hydrophobicity of strain p52 with different carbon sources during 48 h was determined using BATH (Figure 3b). These findings showed that the substrate used by strain p52 had a strong influence on its hydrophobicity. According to the hydrophobicity classification criteria [53], when the concentrations of DF and NaOAc were high, strain p52 was in the strongly hydrophobic (≥50%) and non-hydrophobic (<20%) stage, respectively. This deduction was also confirmed by contact angle measurements; here, strain p52 had a contact angle of approximately 110° (Figure 3c) with water when DF was used as a carbon source and a contact angle of approximately 78.6° (Figure 3d) with water when NaOAc was used as a carbon source after 24 h of cultivation. The presence of DF, as a hydrophobic compound, induced strain p52 to increase its hydrophobicity and facilitated contact and adhesion between strain p52 and DF, thus promoting DF degradation by strain p52. A study using *P. putida* to degrade naphthalene also showed that the bacterial surface hydrophobicity was higher during biodegradation; therefore, the adhesion to naphthalene increased [54]. Interestingly, in the later stages of the culture, the difference in hydrophobicity of strain p52 decreased when DF and NaOAc were used as carbon sources, and they were all at the moderately hydrophobic stage (20%–50%). This was likely caused by the depletion of the carbon source, and the hydrophobicity of strain p52 returned to the initial state. It is notable that this study was conducted under laboratory conditions. There may be some variation during field remediation due to the wide variety of carbon sources. When the concentration of hydrophobic pollutants is high, the strain maintains strong hydrophobicity due to ample carbon sources, which allows pollutants to be degraded efficiently. When the hydrophobic pollutant concentration is low, the hydrophobicity of bacteria is influenced by other carbon sources. If the carbon sources are hydrophobic, the hydrophobicity of bacteria can be maintained at a high level; however, if the carbon sources are hydrophilic, the bacterial surface hydrophobicity may follow a similar trend to that shown by using NaOAc as the carbon source in this study. The former does not disturb the removal of pollutants, but the latter is complex, as the removal rate of the pollutant may change along with hydrophilic carbon source utilization [55]. Thus, a pilot study is needed to further examine the hydrophobicity variation in bacteria during remediation.

### 3.3. Analysis of the EPS Yield of Strain p52

Changes in the biofilm and surface characteristics affect the adhesion of strain p52 to DF, and this is closely related to the EPSs secreted by strain p52. Therefore, the components and content of EPSs were analyzed after 24 h of incubation in DF-supplemented and NaOAc-supplemented CFMM. The selection of this time point was reasonable because the biofilm yield was the highest at 24 h. In this study, *Rhodococcus* sp. was used to degrade 500 mg/L DF, and 132.91 mg/g DW EPS was produced by the bacterium (Table S1). In a previous study, *Chryseobacterium* sp. was used to degrade 30 mg/L DF, and the EPS yield was 21.59 mg/g DW [56]. The EPS yield is influenced by the concentration of carbon sources and related to microbial species. In addition, the present study has only focused on the EPS yield of a single strain in the process of degrading pollutants. A mixed microbial consortium can enhance the yield of EPSs. A previous study has demonstrated that the EPS yield of a mixed bacteria consortium was greater than that of a mono strain when degrading diesel, which was beneficial to the microbial adhesion to and degradation of pollutants [57]. The roles of EPSs in the degradation of DF by mixed microbial flora should be further studied in the future.

**Figure 4:**
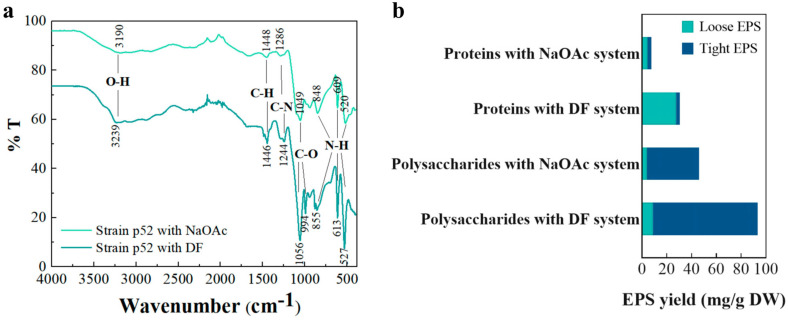
Functional group analysis (**a**) and yield (**b**) of *Rhodococcus* sp. strain p52 EPSs after 24 h (DF: dibenzofuran; NaOAc: sodium acetate; EPS: extracellular polymeric substance).

The EPS yields produced by strain p52 using different carbon sources are shown in Table S1. The results indicate that the yield of EPSs produced by strain p52 was clearly higher when DF was used as the carbon source, as opposed to sodium acetate. The contents of the extracellular polysaccharides and proteins of strain p52 accounted for more than 90% of the total EPS yield in both media, and the yield of extracellular polysaccharides produced by strain p52 was greater than that of proteins with two carbon sources. Our results were validated by previous studies on the EPSs of *Rhodococcus.* Sivan et al. [58] used *R. ruber* to degrade polyethylene and reported that the strain secreted 2.5 times more extracellular polysaccharides than extracellular proteins. To further verify the EPS composition, the functional groups of EPSs were determined using FTIR (Figure 4a). The band at 3100~3300 cm^−1^ was assigned to the –O–H– peaks and indicated the presence of an alcohol group. The presence of the saturated hydrocarbons was indicated by the –C–H– stretching bands of the –CH_3_ and –CH_2_ in the region of 1300~1400 cm^−1^. The 1200~1300 cm^−1^ peak was attributed to the presence of –C–N– functional groups of amide or amine groups, the 900~1100 cm^−1^ peak indicated the presence of –C–O– functional groups of sugars and aromatics, and the peaks in the range of 500~900 cm^−1^ were attributed to the presence of –N–H– functional groups of primary amines. The FTIR results further indicated that the EPSs produced by strain p52 with two carbon sources were mainly polysaccharides and proteins.

To analyze the role of the tight EPS and loose EPS in the adhesion of strain p52 to DF, the distributions of extracellular polysaccharides and proteins in the tight EPS and loose EPS were analyzed (Figure 4b). The polysaccharides produced by strain p52 under the two culture conditions were primarily tight polysaccharides firmly attached to the surface of the bacterium. In DF-supplemented CFMM, the proteins were mostly loose proteins that could be easily separated; interestingly, the yields of the loose proteins and tight proteins were almost the same when NaOAc was used as the carbon source.

When DF was used as a carbon source, the total polysaccharide yield of strain p52 was increased twofold with respect to NaOAc and a corresponding nearly twofold increase in both tight and loose polysaccharides. Weathers et al. [33] reported that the *R. jostii* strain RHA1 secreted more extracellular polysaccharides in the presence of perfluoroalkyl acids and suggested that this correlated with an increase in cellular stress in the presence of perfluoroalkyl acids. Zhang et al. [59] reported that both tight and loose polysaccharides on the cell surface contained hydrophilic and hydrophobic groups that increased the solubility of phenanthrene in water, thereby increasing the mass transfer rate of phenanthrene. Our results were consistent with their understanding that the presence of DF increased the cellular stress of strain p52, which in turn led to the secretion of more extracellular polysaccharides. Both tight and loose polysaccharides produced by strain p52 increased the solubility of DF in water and thus promoted the degradation of DF.

Based on the analysis of the extracellular protein content of the bacteria compared with NaOAc-supplemented CFMM, strain p52 cultured in DF-supplemented CFMM clearly secreted more extracellular proteins, and the increase was found mainly in loose proteins; however, the tight proteins did not change. Yu et al. [60] found that the extracellular enzymes were mainly distributed in the loose EPS. Furthermore, researchers found that bacteria tended to temporarily store low-bioaccessible substances in EPSs, and proteins played an important role in this process. They could reduce the exposure of bacteria to harmful substances [61]. Their study is useful in understanding the reason that the tight proteins and loose proteins do not increase in proportion, similar to polysaccharides. Cellular stress was applied to strain p52 with DF to promote the secretion of more enzymes to alleviate the adverse effects of DF on cells. Moreover, an increase in the loose protein content was beneficial for adhering to and temporarily storing DF for bacterial catabolism.

### 3.4. Effect of EPSs on DF Degradation by Strain p52

**Figure 5:**
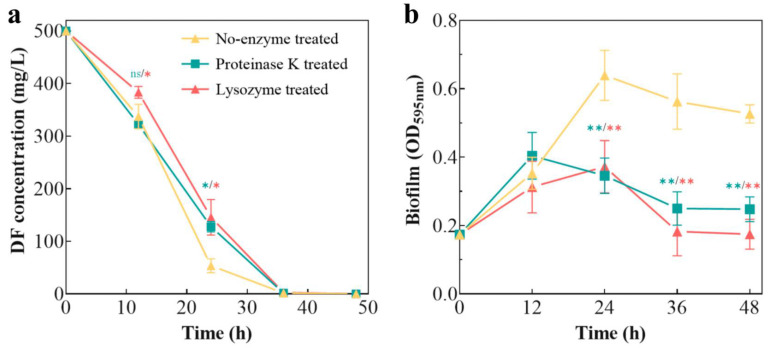
Effect of proteinase K and lysozyme treatment on *Rhodococcus* sp. strain p52. (**a**) Dibenzofuran (DF) degradation. (**b**) Biofilm yield formed by strain p52. Significant differences between the test and control groups are indicated by asterisks at non-significant (ns), *p* < 0.05 (*) and *p* < 0.01 (**).

To verify the importance of EPSs for DF degradation, proteinase K and lysozyme were used to treat strain p52. The degradation efficiency of DF (Figure 5a) and the biofilm yield (Figure 5b) of the enzyme-treated strain p52 were subsequently measured.

Compared with that of the non-enzyme-treated control, the biodegradation efficiency of DF decreased when strain p52 was treated with proteinase K and lysozyme. As shown by the results of the biofilm yield after treatment with proteinase K and lysozyme, the biofilm formed by strain p52 was similarly slumped. The destruction of the polysaccharides and proteins by enzyme treatment indicated that the EPSs secreted by strain p52 affected not only the biofilm formation but also the DF degradation. This finding was consistent with that of Sheng et al. [62], who reported that EPSs secreted by the cells helped bacteria to adhere to and dissolve hydrophobic organic matter, thereby promoting the utilization of the hydrophobic substances. Moreover, EPSs not only trapped the toxic substances in the biofilms and acted as a buffer zone between the toxic substances and bacteria but also increased the adhesion of hydrophobic organic matter, thus improving the biodegradation efficiency [63]. We also found that the biofilm yield under lysozyme treatment was lower than protease K treatment, and lysozyme treatment had a worse effect on the DF degradation efficiency than protease K treatment. This result was also consistent with the higher polysaccharide content in EPSs produced by strain p52 compared with the protein content (Table S1). In this study, after the pre-hydrolysis of extracellular proteins and polysaccharides with proteinase K and lysozyme, the extracellular protein and polysaccharide yield was reduced and the biofilm formation process of strain p52 was blocked; this resulted in a decrease in the degradation efficiency of DF by the enzyme-treated strain p52.

### 3.5. Analysis of the EPS Composition and Proportion of Strain p52

**Figure 6:**
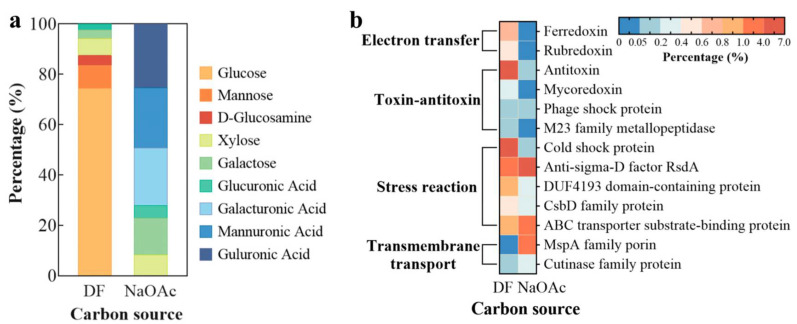
(**a**) Monosaccharide and (**b**) protein composition and proportion of *Rhodococcus* sp. strain p52 EPS at 24 h (DF: dibenzofuran; NaOAc: sodium acetate; EPS: extracellular polymeric substance).

The monosaccharide composition and its proportion in EPSs were determined using ion chromatography and calculated by comparing the spectrum with that of the standard sample. As shown in Figure 6a, the composition and proportion of extracellular polysaccharides greatly varied with the carbon source. When DF was used as a carbon source, the monosaccharide comprising the extracellular polysaccharide of strain p52 was glucose (74.25%); however, when NaOAc was used as the carbon source, glucose was not detected, and the main components were guluronic acid (25.34%), mannuronic acid (24.09%), and galacturonic acid (22.65%). Fakhruddin et al. [64] reported that when the pH was controlled at pH 7.0, the exogenous addition of glucose enhanced the removal of 2-chlorophenol by *P. putida* CP1, because polysaccharides could promote the growth of the strain and induce the formation of NADH and NADPH, which are important cofactors of the pollutant-metabolizing enzymes. That is, glucose not only helped to adhere to and dissolve DF but also promoted the degradation of DF. When NaOAc was used as a carbon source, the polysaccharide produced was mostly viscous uronic acid, which is the main component of mucus substances such as gum, pectin, and bacterial polysaccharides [15]. The production of mucus polysaccharides can promote the closer aggregation of strain p52, just like in the SEM image of the biofilm (Figure 2c).

The extracellular proteins of strain p52 were analyzed via proteomics (Figure 6b). The detected proteins included cytoplasmic proteins, which were released into EPSs through the general secretory pathways and the membrane vesicle release [65]. The proteins related to the electron transfer, toxin–antitoxin, and stress response accounted for a greater proportion of the total protein when DF was used as the carbon source (with the exception of anti-sigma-D factor RsdA); however, the proteins related to the transmembrane transport accounted for a greater proportion of the total protein in the NaOAc-supplemented CFMM. Based on the analysis of the protein content with more than 0.5% under two carbon sources, among the electron transfer proteins, ferredoxin was more predominant in the DF-supplemented CFMM. According to reports, ferredoxin can transfer electrons to dioxygenases, which contributes to the subsequent degradation of DF [66]. Due to the high toxicity of DF to strain p52, the proportion of antitoxin protein in DF-supplemented CFMM was five times greater than that in NaOAc-supplemented CFMM. The percentages of cold shock proteins and DUF4193 domain-containing proteins, which are associated with the stress response, also increased with DF as a carbon source. This phenomenon was also reported in the literature by others. In a study on the dioxin-degrading bacterium *S. wittichii* strain RW1, researchers reported that dioxin degradation led to an upregulation of the cold shock protein expression; they suggested that this upregulation was related to the increased cellular stress caused by dioxin [21]. The DUF4193 family protein is reportedly responsive to redox stress in *M. tuberculosis* [67]. Moreover, the proportion of anti-sigma-D factor RsdA inhibited the stress response of the sigma-D factor [68], decreased twofold in the DF-supplemented CFMM. When NaOAc was used as a carbon source, the transmembrane MspA family pore protein, which mediates the exchange of hydrophilic solutes, was increased fivefold with respect to DF as carbon source [69].

**Figure 7:**
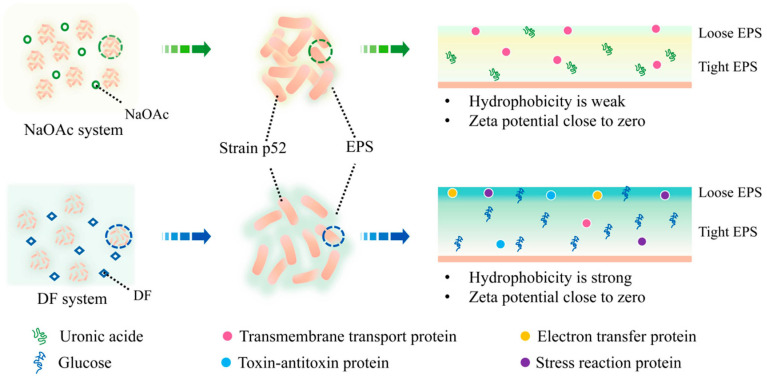
Proposed molecular mechanisms of the efficient adsorption of DF by *Rhodococcus* sp. strain p52 (DF: dibenzofuran; NaOAc: sodium acetate; EPS: extracellular polymeric substance).

The above analyses aimed to explain the roles of EPS content and components in the efficient adhesion of DF by strain p52 (Figure 7). With the catabolism of NaOAc, EPSs were produced with polysaccharides and proteins as the main components. The content of the tight polysaccharides (42.04 mg/g DW) was greater than that of loose polysaccharides (3.90 mg/g DW); however, the content of the tight proteins (3.13 mg/g DW) and loose proteins (4.41 mg/g DW) were similar. Analysis of the polysaccharide and protein compositions revealed that viscous uronic acid accounted for the vast majority of the polysaccharides (72.08%), and the protein related to transmembrane transport was more abundant in the NaOAc-supplemented CFMM than in the DF-supplemented CFMM. Uronic acid in EPSs helps strain p52 to stack more tightly together in the biofilm. Both the rapid decrease in the absolute value of the zeta potential and the hydrophobicity of the strain favored the utilization of NaOAc by strain p52.

Compared with NaOAc as a carbon source, more proteins and polysaccharides were secreted in the DF-supplemented CFMM. The content of both the tight and loose polysaccharides increased by nearly twofold. Interestingly, only the number of loose proteins increased by nearly sixfold in the DF-supplemented CFMM, and the tight protein content did not change. Analysis of the polysaccharide and protein compositions revealed changes. When DF was used as the carbon source, the extracellular polysaccharide was mainly glucose (74.25%), and the proteins related to the electron transfer, toxin–antitoxin, and stress response accounted for a greater proportion than the NaOAc-supplemented CFMM. These compounds could better protect strain p52 and promote the degradation of DF. Because EPSs were an important component of the biofilms, the increase in EPS production also led to an increase in biofilm yield when DF was used as a carbon source. Due to the low percentage of uronic acid and high percentage of glucose in the DF-supplemented CFMM, the biofilms of strain p52 were loose and exhibited porous aggregation; this clustering increased the contact area with DF. Strain p52 has difficulty utilizing the water-insoluble DF, and strain p52 will slowly come into contact with the DF to protect itself. EPSs secreted during the degradation process also reduce the absolute value of the zeta potential, increase the hydrophobicity of the strain p52 surface, and temporarily store the unused DF. This process contributes to adhesion between strain p52 and DF. Although strain p52 secretes EPSs and changes surface characteristics to better adhere to and degrade DF, the utilization efficiency of strain p52 on DF was still not as good as that of NaOAc.

## 4. Conclusions

In this study, the adhesion behavior of strain p52 to DF was elucidated, and evidence showed that EPSs play an important role in this adhesion process. That is, the components and content of polysaccharides and proteins in the EPS were closely related to the adhesion process. Specifically, compared with NaOAc as a carbon source, the elevated percentages of glucose and protein related to the electron transfer, toxin–antitoxin, and stress response, along with the increased production of polysaccharides and proteins, all contributed to the alleviation of cellular stress by strain p52 and the promotion of adhesion between strain p52 and DF. Moreover, biofilm analysis confirmed that the change in EPS components and content increased the biofilm yield and promoted loose and porous aggregation between the bacteria. This aggregation was beneficial for increasing the specific surface area of contact with DF. In addition, the production of EPSs reduced the absolute value of the zeta potential and increased the hydrophobicity of strain p52, which was also beneficial for the adhesion of strain p52 and DF. These findings provide new insight into the adhesion mechanism between DF and DF-degrading bacteria and indicated that the adhesion between strain p52 and DF promoted degradation. Further research is warranted to increase the degradation efficiency of strain p52 by using signaling molecule regulation and mixed carbon source culture and to explore the feasibility of strain p52 for the remediation of real environments. By enhancing the degradation ability of degrading bacteria, we can potentially address the challenge of polycyclic aromatic hydrocarbon and heterocyclic polycyclic aromatic hydrocarbon pollution.

## Data Availability

Data are contained within the article or Supplementary Materials.

## Supplementary Materials

The following supporting information can be downloaded at: https://www.mdpi.com/article/10.3390/microorganisms13010093/s1, Text S1: Measurement of the cell membrane yield; Text S2: Measurement of the hydrophobicity of strain p52; Text S3: Analysis of the monosaccharide composition and content of EPSs; Text S4: Analysis of the protein composition of EPSs. Figure S1: Determination of the maximum absorption wavelength of 1 g/L sodium acetate; Figure S2: Biofilm yield of *Rhodococcus* sp. strain p52 with DF and DD as carbon sources at 24 h (DF: dibenzofuran; DD: dibenzo-p-dioxin). Table S1: Proportions of polysaccharides and proteins in the extracellular polymeric substances (EPSs) of strain p52 with different carbon sources.
