# Supplementary material for: Characterizing the Contaminant-Adhesion of a Dibenzofuran Degrader Rhodococcus sp
Source: Microorganisms. 2025 Jan 6;13(1):93. doi: 10.3390/microorganisms13010093 (PMC11767811; doi:10.3390/microorganisms13010093)
Supplement: Supplementary file 1 [file microorganisms-13-00093-s001.zip › microorganisms-3389874-supplementary.pdf]

Supplementary Information

Characterizing contaminant-adhesion of a dibenzofuran degrader *Rhodococcus* sp.

Yu Chen [1], Qingquan Wei [1], Xudi Wang [1], Yanan Wu [1], Changai Fu [1], Xu Wang [1], Hangzhou Xu [1,2,*] and Li Li [1,*]

[1] Shandong Provincial Key Laboratory of Water Pollution Control and Resource Reuse, School of Environmental Science and Engineering, Shandong University, Qingdao, China, 266237;

[2] Shandong Provincial Engineering Center on Environmental Science and Technology, Jinan, China, 250061

* Correspondence: sdxuhangzhou@163.com (H.X.); lili@sdu.edu.cn (L.L.);

4 Texts

2 Figures

1 Table

Text S1. Measurement of the cell membrane yield

Crystal violet staining was used to measure the cell membrane yield. The cell culture broth was removed and the cell were washed with CFMM, immobilized in anhydrous methanol and dyed with 200 μL crystal violet for 30 min, Afterwards, 200 μL of acetic acid was added to each well and the absorbance at 595 nm was measured.

Text S2. Measurement of the hydrophobicity of strain p52

Bacteria adherence to hydrocarbons method (BATH) was used to hydrophobicity measurement. The cells absorbance (A_0) was measured at 400 nm. Subsequently, 0.2 mL of n-hexadecane was added to 1.5 mL bacterial suspension, vortexed for 3 min to mix the aqueous and organic phases, and stood for 1 h. Absorbance (A_1) was measured at 400 nm.

$$\text{Cell surface hydrophobicity (\%)} = [(A_0 - A_1)/A_0] \times 100 \quad (1)$$

Text S3. Analysis of the monosaccharide composition and content of EPS

Approximately 5 mg of sample was hydrolyzed with trifluoroacetic acid (2 M) at 105 °C for 6 h in a sealed tube. Dry the sample with nitrogen. Add methanol to wash, then blow dry, repeat methanol wash 2-3 times. The residue was re-dissolved in deionized water and filtered through 0.22 μm microporous filtering film for measurement.

The sample extracts were analyzed by high-performance anion-exchange chromatography (HPAEC) on a CarboPac PA-10 anion-exchange column (4.6 × 250 mm; Dionex) using a pulsed amperometric detector (PAD; Dionex ICS 5000

system). Flow rate, 0.5 mL/min; injection volume, 5μL; solvent system A: (ddH_2O), solvent system B: (0.1M NaOH), solvent system C: (0.1M NaOH, 0.2M NaAc); gradient program, 95:5 V/V at 0 min, 80:20 V/V at 30 min, 60:40 V/V at 30.1 min, 60:40 V/V at 45min, 95:5 V/V at 45.1 min, 95:5 V/V at 60 min.

Text S4. Analysis of the protein composition of EPS

The lysis buffer was added to the sample (1.5% SDS/100 mM Tris-HCl, pH=8.5). The sample was then smashed with a t issue homogenizer and incubated at 95°C for 15 min to complete protein denaturation. After the sample cooled down, ultrasonic treatment and centrifuge were performed. The precipitation was resuspended in redissolved solution (8 M Urea/100 mM Tris-HCl, pH = 8.5). After the protein precipitation was redissolved, the protein concentration was determined by BCA method. With the same amount of protein among different samples, the redissolved solution was used to fill all samples to the same volume. Protein reduction and alkylation were conducted with TCEP and CAA at 37°C for 1 h. Urea was diluted below 2 M using 100 mM Tris-HCl. Trypsin was added at a ratio of 1:50 (enzyme:protein, w/w) for overnight digestion at 37°C. The next day, TFA was used to bring the pH down to 6.0 to end the digestion. After centrifugation (12000 g, 15 min), the supernatant was subjected to peptide purification using a self-made SDB-RPS desalting column. The peptide eluate was vacuum dried and stored at -20°C for later use.

All samples were analyzed on an UltiMate 3000 RSLCnano system coupled

on-line with Q Exactive HF mass spectrometer t hrough a Nanospray Flex ion source. Peptide samples were injected into a C18 Trap column (75 μm × 2 cm, 3 μm particle size, 100 Å pore size, Thermo), and separated in a reversed-phase C18 analytical column packed in-house with ReproSil-Pur C18-AQ resin (75 μm × 25 cm, 1.9 μm particle size, 100 Å pore size). Mobile phase A (0.1% formic acid/3% DMSO/97% H_2O) and mobile phase B (0.1% formic acid/3% DMSO/97% ACN) were used to establish the separation gradient at a flow rate of 300 nL/min. The MS was operated in DDA top 20 mode with a full scan range of 350-1500 m/z. AGC Target value for the full MS scan was 3E6 charges with a maximum injection time of 30 ms and a resolution of 60,000 at m/z 200. Precursor ion selection window was kept at 1.4 m/z and fragmentation was achieved by higher-energy collisional dissociation (HCD) with a normalized collision energy of 28. Fragment ion scans were recorded at a resolution of 15,000, an AGC of 1E5 and a maximum fill time of 50 ms. Dynamic exclusion was enabled and set to 30 s.

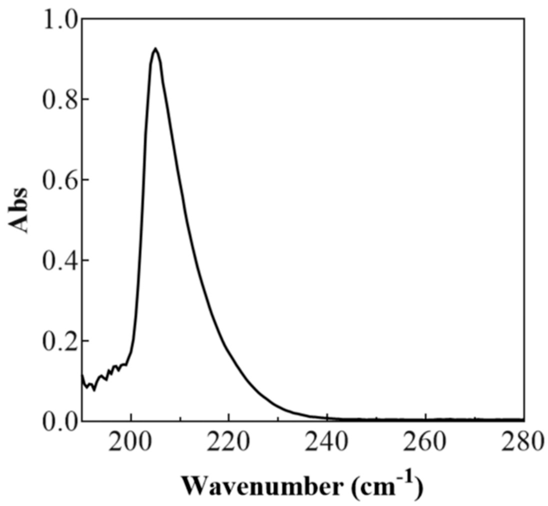

Figure S1. Determination of the maximum absorption wavelength of 1 g/L sodium acetate.

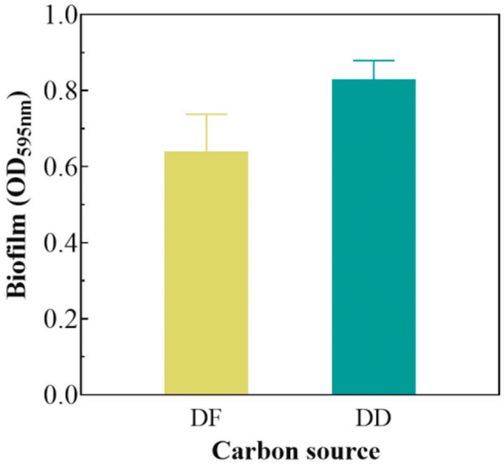

Figure S2. Biofilm yield of *Rhodococcus* sp. strain p52 with DF and DD as carbon sources at 24 h (DF: dibenzofuran; DD: dibenzo-*p*-dioxin).

Table S1. Proportions of polysaccharides and proteins in the extracellular polymeric substances (EPS) of strain p52 with different carbon sources.

Carbon source	Total EPS (mg/g DW)	Polysaccharides (%)	Proteins (%)
DF	132.91	70.27	23.10
NaOAc	59.17	77.64	12.74
